# Supplementary material for: Netcombin: An algorithm for constructing optimal phylogenetic network from rooted triplets
Source: PLoS One. 2020 Sep 18;15(9):e0227842. doi: 10.1371/journal.pone.0227842 (PMC7500971; doi:10.1371/journal.pone.0227842)
Supplement: S4 File — (ZIP) [file pone.0227842.s004.zip › SequenceData-Triplet/sequenceData-10-20-30-0-Triplet/length 500, numseq 30, rate 10^-7/3 seq.pdf]

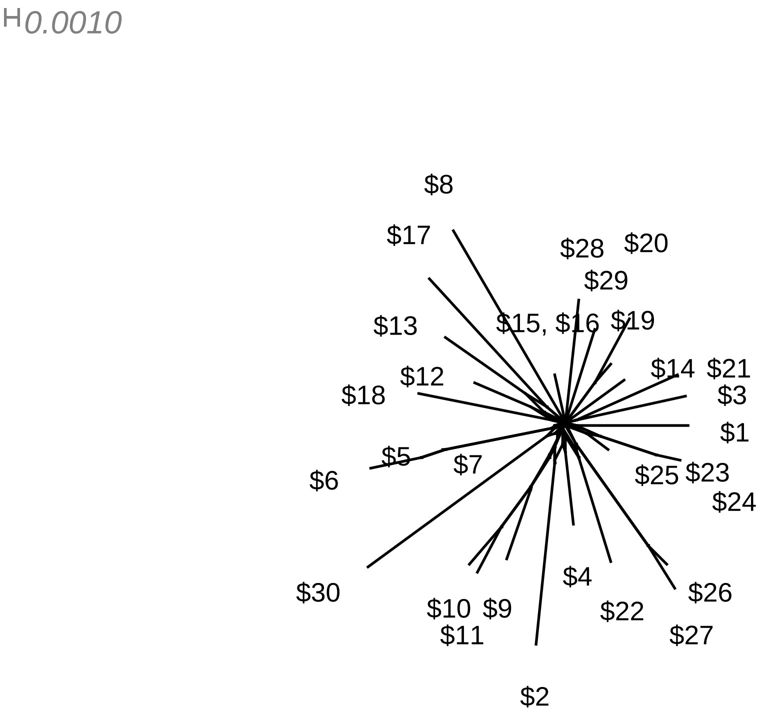
0.0010
$8
$17
$28
$20
$29
$13
$15, $16
$19
$14
$21
$12
$3
$18
$1
$5
$7
$25
$23
$6
$24
$30
$4
$26
$10
$9
$22
$11
$27
$2
